# Multiple instance learning detects peripheral arterial disease from high-resolution color fundus photography

**DOI:** 10.1038/s41598-022-05169-z

**Published:** 2022-01-26

**Authors:** Simon Mueller, Maximilian W. M. Wintergerst, Peyman Falahat, Frank G. Holz, Christian Schaefer, Nadjib Schahab, Robert P. Finger, Thomas Schultz

**Affiliations:** 1 B-IT and Department of Computer Science, University of Bonn, 53115 Bonn, Germany; 2 Department of Ophthalmology, University Hospital Bonn, 53127 Bonn, Germany; 3 Department of Cardiology and Angiology, University Hospital Bonn, 53127 Bonn, Germany

**Keywords:** Peripheral vascular disease, Computer science, Retina, Diagnostic markers, Machine learning

## Abstract

Peripheral arterial disease (PAD) is caused by atherosclerosis and is a common disease of the elderly leading to excess morbidity and mortality. Early PAD diagnosis is important, as the only available causal therapy is addressing risk factors like smoking, hypercholesterolemia or hypertension. However, current diagnostic techniques often do not detect early stages of PAD. We theorize that PAD’s underlying cause atherosclerosis can be detected on color fundus photography (CFP) images with a convolutional neural network architecture, which might aid earlier PAD diagnosis and improve disease monitoring. In this explorative study a deep attention-based Multiple Instance Learning (MIL) architecture is used to capture retinal imaging biomarkers on CFP images of 135 examinations. To capture subtle variations in vascular structures, higher image resolution can be utilized by partitioning the CFP into patches. Our architecture converts each patch into a feature vector, and determines its relative importance via an automatically computed attention weight. Our best model achieves an ROC AUC score of 0.890. Visualizing these attention weights provides insights about the network’s decision and suggests ocular involvement in PAD. Statistical analysis confirms that the optic disc and the temporal arcades are weighted significantly higher (p < 0.001) than retinal background. Our results support the feasibility of detecting the presence of PAD with a modern deep learning approach.

## Introduction

Undiagnosed peripheral artery disease (PAD) is common among the elderly population. In a study testing 613 men and women with an average age of 66 years, 11.7% showed signs of large-vessel PAD^1^. Early disease detection is challenging as early stages remain asymptomatic. However, early diagnosis is important to allow for preventive measures avoiding irreversible damage leading to reduced mobility, quality of life and increased mortality^2^. Even asymptomatic patients have a mortality rate twice as high compared to healthy controls^2^.

The disease is caused by atherosclerosis, plaque build-ups in the peripheral arteries interfering with the normal circulation and in turn with local perfusion. Later stages of PAD can severely limit or completely block the blood flow to the affected limb, which can eventually require amputation^3^. As the underlying cause (atherosclerosis) affects all arteries in the body we theorized that vascular alterations are also present in the retinal arterial vessels. In fact, our previous work linked vascular abnormalities on ocular optical coherence tomography angiography (OCT-A) to PAD^4^. In our current work, we aim to investigate whether a similar link can be made with results from color fundus photography (CFP), a widely available and non-invasive imaging modality in which no specific findings characteristic for PAD have been described so far^5^.

In the last couple of years, Convolutional Neural Network (CNN) architectures have shown an ability to solve complex medical image analysis tasks, sometimes relying on subtle imaging biomarkers that are not readily apparent to the human eye. They have also been applied to challenges within ophthalmology. A prominent example of this is a system for the CFP-based detection of diabetic retinopathy which has been certified by the U.S. Food and Drug Administration (FDA) to make recommendations regarding the need for further intervention completely autonomously^6^.

In this proof-of-concept study, we assess the feasibility of using deep learning to detect PAD based on CFP images. Our work differs from a recent CNN-based approach for detecting atherosclerosis from fundus images^7^ in that we use a Multiple Instance Learning (MIL) architecture which allows us to process the images at higher resolutions. We experimentally verify that this substantially increases our model’s accuracy, indicating that the alterations due to atherosclerosis occur at a fine spatial scale. Moreover, the MIL approach involves an attention mechanism whose weights determine which image regions should contribute most to the final prediction. Visualizing these weights indicates that the network mostly relies on the optic disc and the temporal retinal vessels to detect the presence of atherosclerosis.

Section “Materials and methods” will present our data and proposed methods, while section “Results” evaluates the achieved performance, and the importance of processing the image at a high resolution. Section “Discussion” discusses our findings, including an analysis of our model’s weight maps to identify which structures in the fundus images are most important for the detection of PAD.

## Materials and methods

We acquired a novel dataset and annotated it as described in section “Data acquisition and annotation”. We then developed an end-to-end image analysis system using a pre-trained attention-based MIL architecture. An activation map using the original images is generated by using the attention vector of the MIL model (“Data acquisition and annotation”). The system was implemented in Python, using the packages scikit-learn, PyTorch, OpenCV, and Pandas.

### Data acquisition and annotation

Our primary dataset has been acquired at the Department of Ophthalmology, University Hospital Bonn, Germany. Approval was obtained from the ethics committee of the University Hospital Bonn (approval ID 047/18) and informed consent was obtained from all study participants. The study was conducted according to the tenets of the Declaration of Helsinki. Inclusion criteria were a diagnosis of PAD based on clinical staging according to Fontaine and characteristic changes of the arteries of the lower extremities in color-coded Doppler sonography or computer tomography angiography or pathologic ankle-brachial-pressure-index (ABI) according to the current classification of PAD^8^. We excluded all patients with any current ocular symptoms or signs, a history of any ocular surgery (except cataract surgery) or a history of any ocular diseases, corneal, lens or vitreous opacities, or poor CFP image quality. The dataset contains CFP images from 135 eyes that have been acquired with an EIDON widefield TrueColor confocal fundus imaging system (CenterVue, Padova, Italy). Per patient and per eye one photograph was recorded with an angle of retinal view of 120 degrees. The assessment of PAD was performed manually by measurement of the ABI and the Fontaine classification^9^. All measurements were performed with the use of appropriately sized pneumatic cuffs for both the ankle and the arm. The systolic ankle pressures were recorded with a handheld 5 MHz bi-directional pocket Doppler instrument by continuous wave velocity detection (Bidop ES-100V3, HADECO, Kawasaki, Japan). The class distribution in the dataset is unbalanced, with 59/34/4/0 eyes for Stage I, Stage II, Stage III, and Stage IV in the Fontaine system. The control group is represented by 34 healthy, age-matched examination samples. Additional characteristics of the dataset are presented in Table 1.

**Table 1:** Characteristics of the dataset including age, sex, maximum Fontaine stage and lowest ankle-brachial-index.

	Mean (range) ± SD or n (%)	
	PAD	Controls
Age	67.94 ± 9.47 (46 - 89)	73.75 ± 8.02 (46–89)
Sex (male)	43 (75%)	9 (45%)
Lowest ankle-brachial-pressure-index	0.92 ± 0.32 (0.16–1.49)	–
History of acute coronary syndrome	32 (28%)	0
**Maximum Fontaine stage**		
I	59 (61%)	0
IIa	8 (8%)	0
IIb	26 (27%)	0
III	4 (4%)	0
IV	0	0

The photographs are centered on the macula and have a resolution of $3680\times 3288$ pixels. The images underwent no further preprocessing after the acquisition and thus contain varying lighting conditions and rotations. The dataset is split into 7 folds using stratified sampling. The folds are stratified so that, to the extent possible, they preserve the percentage of samples for each of the above-mentioned five classes.

To enable a statistical analysis of how attention weights depend on various anatomical structures, we manually annotated the macula, the optic disc, the superior and inferior nasal and temporal arcades of the retina in all images. Figure 5a shows an example of this annotation. Annotations were performed with the software VGG Image Annotator (VIA)^10^ in the form of tight bounding polygons around the respective structures.

For pre-training our CNN architecture, we collected additional data from the diabetic retinopathy detection Kaggle challenges in 2015 and 2019^11–13^. We split the images for pre-training into a training set of 83126 samples, and a validation set of 9237 samples. For preprocessing, these images are resized to $1024\times 1024$ pixels and cropped to a circle. Black borders are also detected and removed. We emphasize that this re-scaling has only been done on the data used for pre-training, to unify the different resolutions stemming from a multitude of different medical imaging devices. Images in our primary dataset have been processed at their native resolution.

### Classification of peripheral artery disease

Most standard CNN architectures for image classification require input images at a fixed and relatively small resolution. Prior work on the detection of atherosclerosis in retinal images has met this requirement by down-scaling the fundus images^7^. We hypothesized that such an approach can lead to insufficient detection of small details, since reducing the resolution makes small changes around the vessels and finer vascular structures invisible, both to the CNN and to the human eye. Therefore, we decided to instead partition the image into patches each of which shows a small region within the retina. This allows us to work with the retinal photographs at their original resolution.

**Figure 1:**
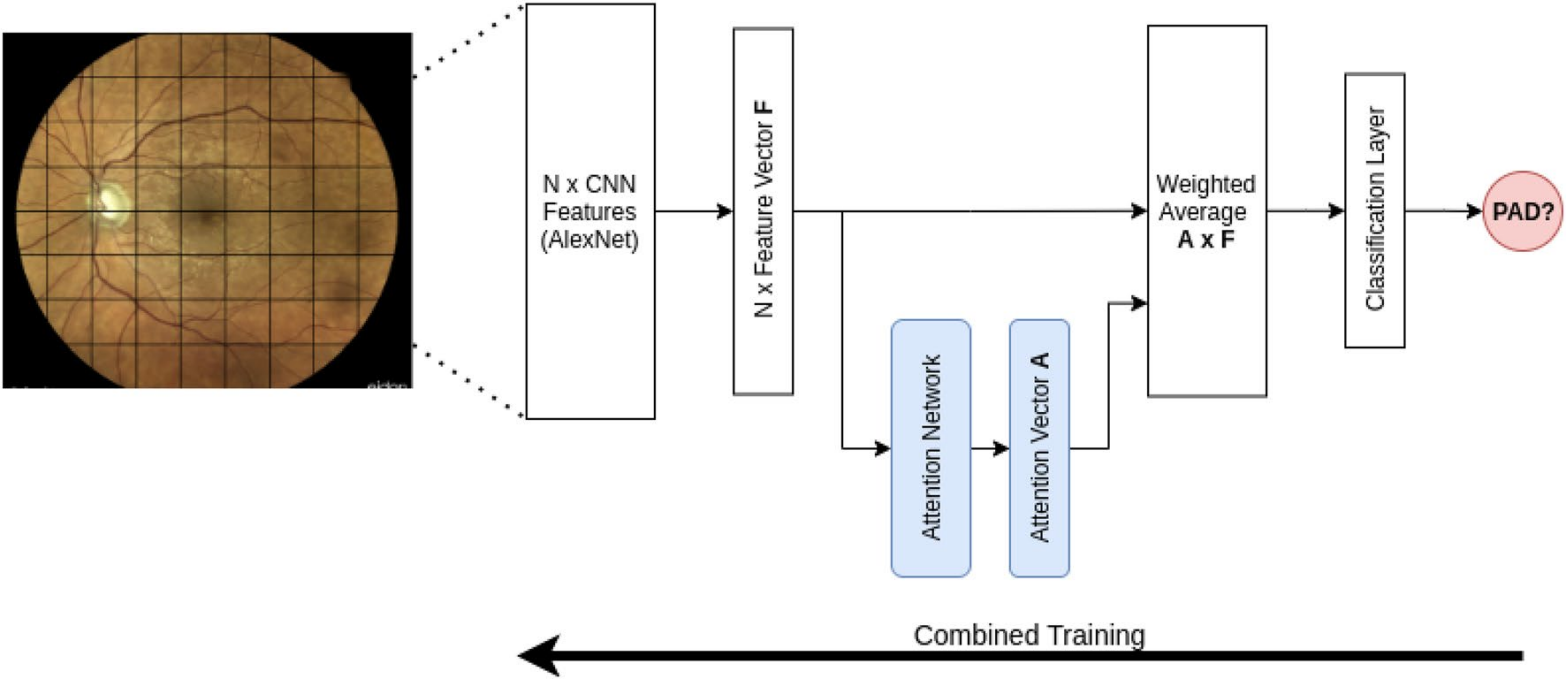
Overview of the attention-based multiple instance learning model. The high-resolution image is subdivided into N patches, which are transformed into N feature vectors by an AlexNet CNN. They are propagated into an attention network that generates a weight for each patch, reflecting the extent to which it should affect the final decision. Based on the attention values, a weighted average of the N feature vectors is taken. It serves as a basis for the final disease prediction. The attention and feature nets are trained jointly.

After processing the individual image patches with a CNN, the resulting information has to be merged again to arrive at a prediction at the eye or patient level. This information fusion accounts for the fact that different patches might carry a varying amount of information about the disease. In particular, we hypothesized that subtle vascular changes which might be especially relevant in the detection of atherosclerosis, might, however, not be equally visible in all patches. We address this via Multiple Instance Learning (MIL), a weakly supervised approach that can be applied to so-called bags of samples only some of which need to be informative about the target label. In our context, each patch is a sample, the bag contains all patches from an eye or patient, and the target label is the presence of PAD. Specifically, we use the strategy of Ilse et al.^14^, in which an attention mechanism allows the network to learn on which patches it should rely most for its prediction. This approach is illustrated in Fig. 1. It has the additional advantage that the attention weights can be visualized as an overlay on the original image, and that they can be compared statistically between patches showing different anatomical structures. This provides visual and quantitative tests of our hypothesis that vascular structures are most relevant for PAD detection.

We partitioned the retinal images into patches of $399\times 399$ pixels, resulting in 72 patches per eye. We exclude patches at the boundary as less informative if they show more than 50% black pixels. The remaining patches form a bag for the MIL approach. As all images in our primary dataset were recorded on the same hardware and have a similar resolution, the number of patches per bag does not vary substantially. Our implementation of the MIL approach uses an AlexNet^15^ convolutional neural network to extract features ${\mathbf {h}}_k$ from each fundus segment *k* within the above described bag. Based on those features, a second network calculates attention weights $\alpha _k$. The entire bag is then represented through the weighted sum of features ${\mathbf {z}}=\sum \alpha _k{\mathbf {h}}_k$. We note that Ilse et al. also propose a gating mechanism^14^. We do not use it in our work, because it did not improve performance for our dataset in initial tests. Finally, ${\mathbf {z}}$ is fed into a fully-connected layer to obtain a binary classification between the control and the disease class. The disease group includes all four Fontaine stages of PAD.

The MIL network was trained for 50 epochs, using a standard cross-entropy loss, an Adam Optimizer^16^ with an initial learning rate of $10^{-4}$, learning rate reduction on plateau, batch normalization, inverse class frequency weighted sampling, weight decay of $10^{-3}$, and various data augmentation techniques (flipping, affine transformations, RGB-shifts, noise and cutouts). The attention weight network has 128 nodes.

Given that modern CNN architectures have to be trained on large amounts of data to achieve their full potential, it is common to employ transfer learning when the primary dataset itself has a limited size. This means that the network weights are initialized by training the same architecture on another, much larger dataset^17^. This assumes that the weights obtained by such a pre-training step are more similar to ones that provide useful results in the primary image domain compared to a random initialization. As it is commonly done, we initialize the AlexNet with weights from training on the ImageNet dataset^18^. In an earlier work, we found out that further refining the ImageNet based weights using images from Kaggle challenges (as described in “Data acquisition and annotation”) improved results in another ophthalmic medical image analysis task, since those images were more similar to our final task than those from ImageNet^19^. We applied the same pretraining for our PAD dataset: Pre-training was performed on an AlexNet with cross-entropy loss, an Adam optimizer, some data augmentations, initial learning rate of $10^{-4}$ and weight decay of $10^{-3}$.

### Visualizing the network attention

The attention mechanism increases the accuracy of the multiple instance learning approach by allowing it to focus on the image patches that contain the most relevant information. Moreover, it makes the classification more interpretable, since the attention weights can be visualized to highlight the parts of the image that the classifier relied on most. Creating a correct visualization involves mapping the one-dimensional attention vector that is used by the model back to the two-dimensional image. For this, we first insert a zero weight for each of the mostly black patches that had been removed earlier. The resulting vector can be re-shaped into a two-dimensional map whose dimensions match the number of image patches into which the image has been subdivided in vertical and horizontal direction, respectively. For our dataset, this leads to a $8 \times 9$ grid.

We upsample the attention weights to the original image resolution using bilinear interpolation, color code them as a heatmap, and overlay them on the CFP image. The maximum attention weight can vary a lot depending on the input image. To make the visualizations more comparable, the range of the heatmap is fixed so that all attention weights that exceed a pre-specified maximum are mapped to maximal intensity.

### Statistical analysis of the network attention

**Figure 2:**
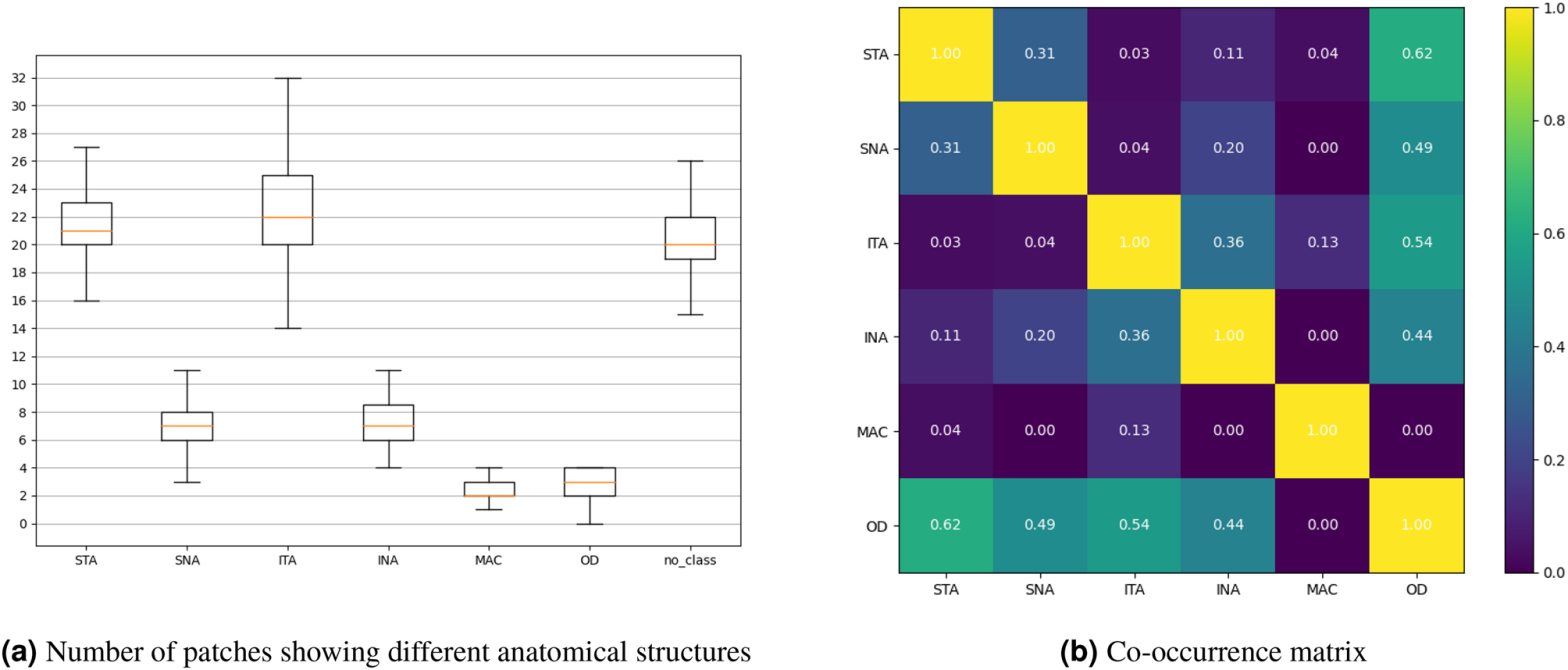
The boxplot diagram (**a**) illustrates the number of image patches for the different anatomical structures across all 135 eye examinations. The co-occurrence matrix of anatomical structures within one patch is displayed in (**b**). When two or more classes are visible in one patch their appearance is counted in the matrix. Next, the matrix is normalized to range between 0 and 1 by an intersection over union norm. Class legend: *S/I* superior/inferior, *T/N* temporal/nasal, *A* arcade, *MAC* macula, *OD* optic disc.

Visual inspection of heatmaps only permits a qualitative assessment of our hypothesis that it is mostly the visual appearance of blood vessels in CFP that permits a detection of peripheral artery disease. Therefore, we also conducted a quantitative statistical analysis of how the network attention weight of a given image patch depends on the anatomical structures that are visible in it. To this end, for each image, we identified the patches overlapping the polygons that were used to annotate the anatomical structures as described in “Data acquisition and annotation”. Patches showing mostly black pixels were removed from the analysis, in the same way in which they were filtered from the MIL network input. This resulted in seven groups, one each for the macula, the optic disc, and the superior and inferior nasal and temporal arcades. A seventh group corresponds to patches that did not contain any annotated structures. In our statistical analysis, we averaged the attention weights across all patches in a group, and tested whether sorting groups based on their average attention ranked some of them significantly higher than others across all 135 images. Figure 2a shows the distribution of the number of patches in each group. Since the network weights were non Gaussian distributed, we selected the Friedman test^20^ to perform an initial check for an overall difference between all seven groups.

Figure 2b illustrates that certain classes, especially the optic disc, frequently occur in the same patch as others. This implies that the corresponding average weights are not independent, which would complicate the interpretation of pair-wise tests. Therefore, we focused our post-hoc analysis on two aspects that did not involve any overlaps: First, we tested, for each of the six anatomical structures separately, whether its average attention weight differed (two-sided) from the patches showing none of the structures. Second, for the temporal and nasal regions combined, we compared the attention weights of patches showing arteries but no veins to those showing veins but no arteries. These comparisons used the Wilcoxon signed-rank statistic^21^ with Bonferroni correction to avoid an inflation of Type I errors due to the multiple tests. With family-wise $\alpha = 0.05$ (“significant”) and $\alpha =0.01$ (“highly significant”), this led to corrected thresholds of $p < 0.00714$ and $p < 0.00142$, respectively.

### Ablation study

Our main motivation for using a multiple instance learning approach is to provide the network with a high input image resolution, so that it can detect atherosclerosis based on fine vessel structures and details. To check whether this high resolution is indeed necessary, we repeat our main classification experiment after reducing the resolution of all images used for training and testing by down-scaling them to $299 \times 299$ pixels, which is a normal input resolution for many CNNs^22,23^. After upscaling them again to the original dimensions ($3680 \times 3288$ px) using bilinear interpolation, we train and test the same attention-based MIL architecture with these modified images. In this ablation study, we expected a significant loss of predictive power because the network will no longer be able to extract relevant details from the low-resolution images. We also test the same assumption for other resolutions (399, 599, 799, 999 and 1299 pixels) and compared the results to the original resolution to find an optimal input size. After proving the usefulness of using the full resolution this ablation studies aims at determining the optimal grade of detail of the input images. By generating difference images (Fig. 6) between the original resolution and the down-sampled photograph we investigate the information loss due to the scaling.

## Results

### Evaluation of disease detection

The following results were observed in the introduced experiments using our dataset of 135 eye examinations. The best attention-based MIL model (see Table 2) utilizes pre-training and CFPs down-sampled to $799 \times 799$ px. The first row reports results from an ablation study for a network not using the Kaggle pre-training. In this scenario only the normal ImageNet weights are used for initialization of the feature extractor network stump. With an ROC AUC-score of 0.653 the performance of this model is much lower than for the fully pre-trained architecture (0.810). This illustrates that the extra step in the pre-training provides a clear advantage for the prediction. Our second ablation study simulates the loss of image resolution that would have occurred when using many of the standard CNN pipelines for image classification. Reducing the resolution to $299 \times 299$ pixels for the full image reduces the attention-based MIL performance drastically to an ROC AUC score of 0.628. In the context of this experiment we determined the best input resolution for the MIL network as an value between 799 and 999 pixels. This adjustment improves the ROC AUC metric from 0.810 to 0.890. Figure 6 shows that edge information is lost by down-scaling to lower resolutions and is conserved for images size greater than 799 px.

**Table 2:** We applied different performance measures to the attention-based MIL classifier. They show a clear benefit from using Kaggle challenge data for pre-training. ROC AUC and PR AUC are the area under the receiver operating characteristic and the precision recall curve, respectively. Searching for the optimal input resolution of the images (values between $299 \times 299$ px and $1299 \times 1299$ px) shows that $799 \times 799$ px produces the best results. Highest ROC AUC over models is highlighted in bold.

	Performance measure					
	Precision	Recall	Accuracy	F1	ROC AUC	PR AUC
Model with ImageNet Pre-Training (Full resolution)	0.788	0.772	0.674	0.78	0.653	0.859
Model with KAGGLE Pre-Training (Full resolution)	0.880	0.802	0.770	0.839	0.810	0.925
Full Model with down-sampled data ($299 \times 299$ px, ablation study)	0.809	0.713	0.659	0.758	0.628	0.840
Full Model ($799 \times 799$ px)	0.954	0.822	0.837	0.883	**0.890**	0.964

**Figure 3:**
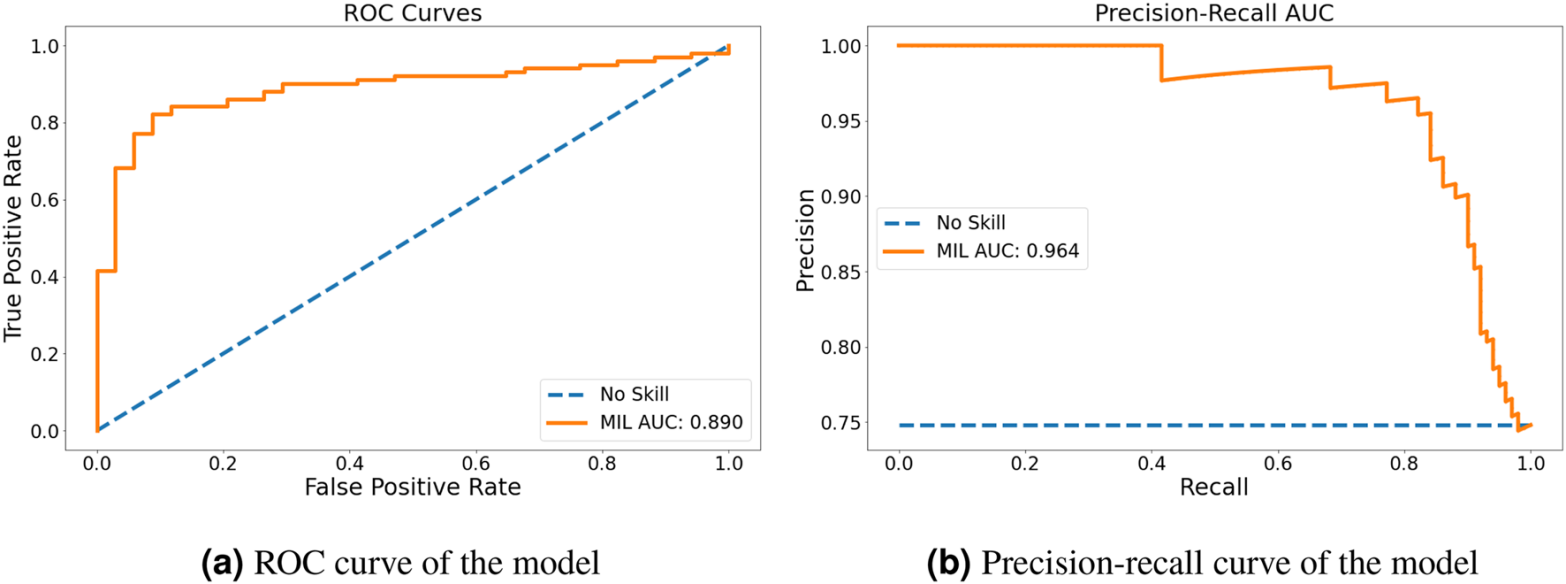
Varying the final probability threshold yields the receiver operator characteristics shown in (**a**) and the precision-recall curve in (**b**). Orange represents our proposed method.

The Receiver Operating Characteristic (ROC) and precision-recall (PR) curves are visualized in Fig. 3. Subfigure (a) highlights that our attention-based MIL approach was able to achieve an AUC ROC value of 0.890. Due to the imbalanced dataset, the AUC of the precision recall curve, shown in Subfigure (b), is even higher (0.964).

**Figure 4:**
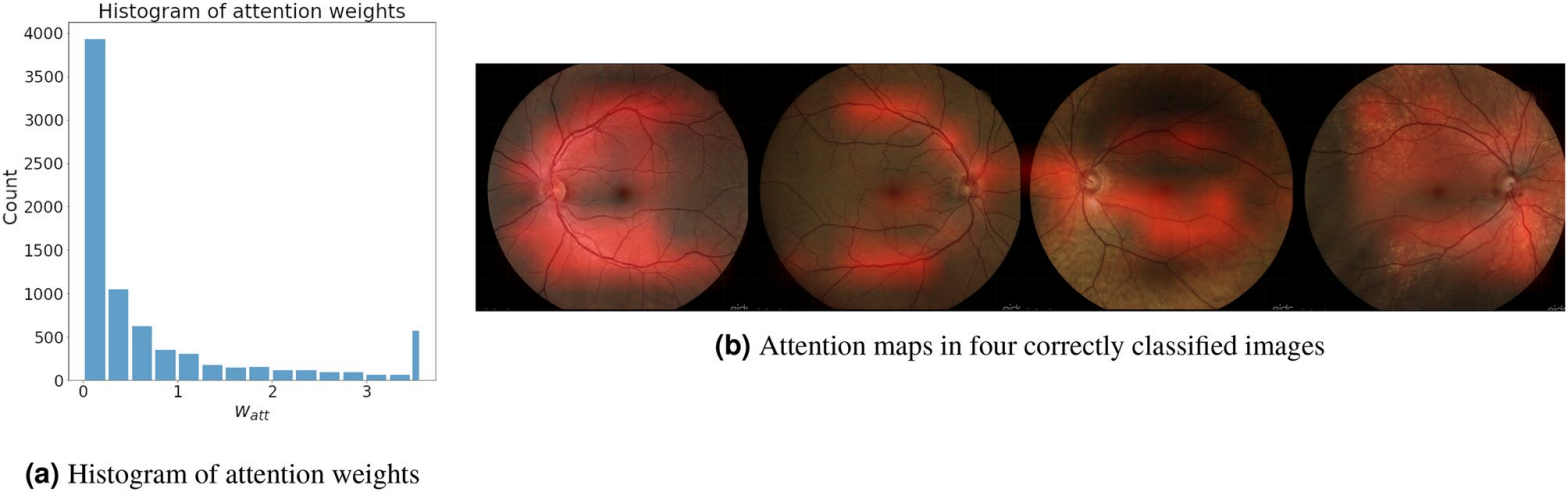
Subfigure (**a**) shows a histogram of all attention weights from the images in the validation set across all k-folds. It indicates that the network ignores many less relevant patches in the input images. All values above 3.5 are combined into the rightmost bar. Attention maps in Subfigure (**b**) correspond to four correct predictions of PAD. The first two images are true positives and the last two true negatives. The maps suggest that the network mostly relied on patches showing retinal vessels for the prediction.

In Fig. 4a the distribution of attention weights is shown as a histogram. Since a few patches received very large weights, the histogram has been truncated at $w_{att}=3.5$. The last bin contains all weights greater than this threshold. A large number of small weights indicates that the network made use of the attention mechanism to ignore many patches that apparently had little value for detecting PAD. In Fig. 4b, the attention maps for four correct predictions are shown. The first two images are true positives and the last two true negatives. The network displays strong attention values in areas around the optic disc and the vasculature.

This qualitative impression is supported by the statistical test results shown in Table 3. The Friedman test indicates a highly significant overall difference between the six anatomical groups and the background. Pairwise comparisons with the Wilcoxon signed-rank test indicate highly significant differences between empty patches, which do not contain any annotated structure, and patches showing the optic disc or the temporal retinal arcades. Differences in attention weights between macular and inferior nasal arcade patches and empty patches were not significant when accounting for Bonferroni correction.

**Table 3:** Results of three statistical tests to investigate the role of specific anatomical structures in the predictions. * and ** mark significant and highly significant p-values ($\alpha = 0.05$ and $\alpha = 0.01$). Bonferroni correction is applied to the Wilcoxon signed-rank tests ($\alpha _{{\rm ind}}=0.00714$ and $\alpha _{{\rm ind}}=0.00142$). The tests compare patches containing anatomical structures with ones that show background.

	Tested structure(s)	N (per group)	p-value
Friedman test	All classes	135	$4.780\times 10^{-09}$**
Wilcoxon signed-rank test	Sup. temporal arcade	135	$1.292\times 10^{-09}$**
	Sup. nasal arcade	135	$1.058\times 10^{-03}$**
	Inf. temporal arcade	135	$2.080\times 10^{-09}$**
	Inf. nasal arcade	135	$2.624\times 10^{-02}$
	Macula	135	$1.684\times 10^{-01}$
	Optic disc	135	$5.390\times 10^{-07}$**
Wilcoxon signed-rank test	Arteries vs. veins	135	$1.416\times 10^{-02}$

**Figure 5:**
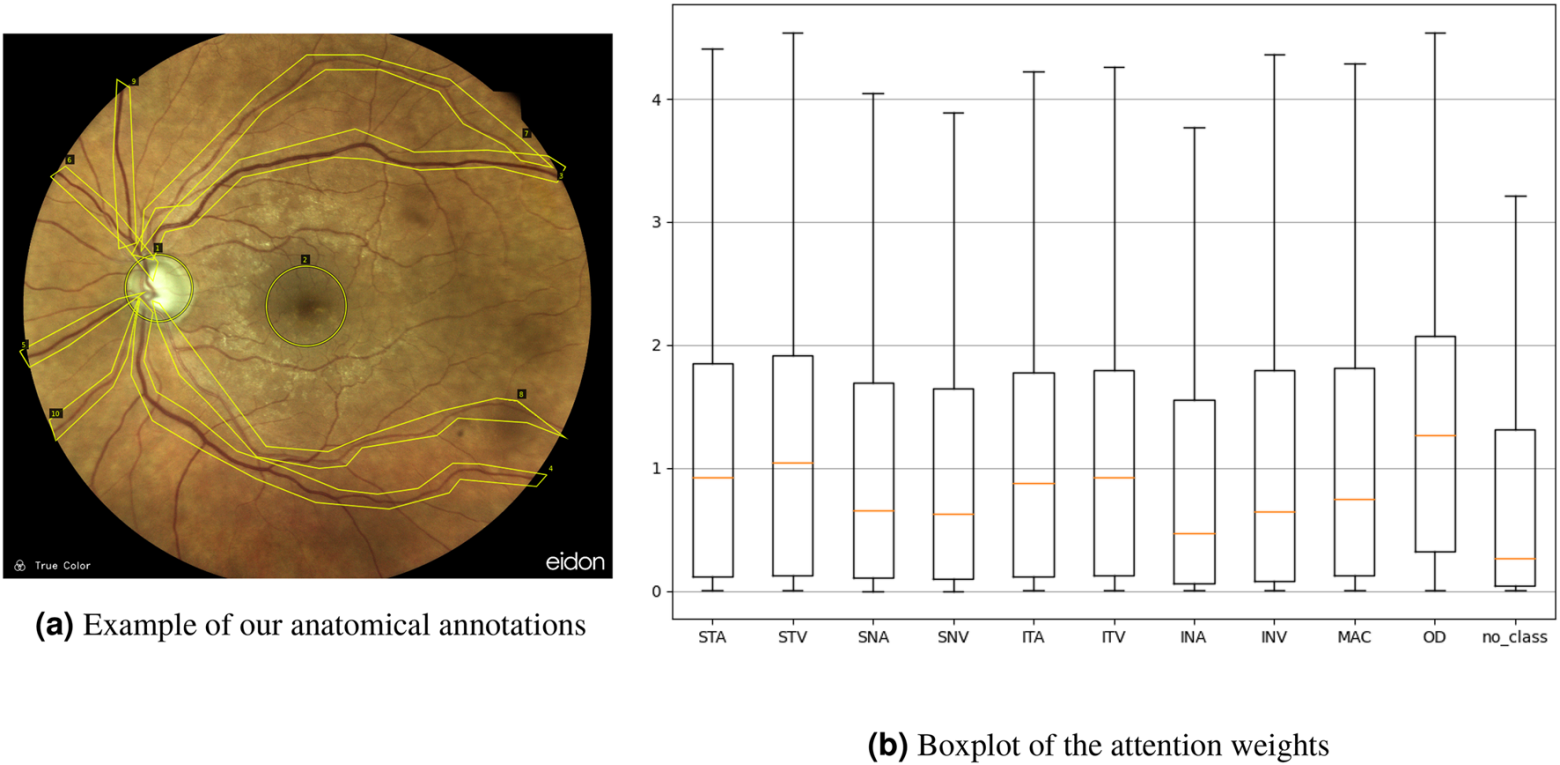
(**a**) Is an example of the different anatomical structures that were annotated. (**b**) Shows boxplots of the attention weights according to the anatomical structure they are showing. Class legend: *S/I* superior/inferior, *T/N* temporal/nasal, *A/V* artery/vein, *MAC* macula, *OD* optic disc.

Figure 5 provides additional insight on the direction and magnitude of differences by plotting the distributions of attention weights for all groups. Median attention weights were highest for the superior and inferior arcades and the optic disc. Corresponding arteries and veins have been combined into a single group in the analysis above. Comparing them shows that veins ($\mu = 1.016 \pm 0.5249$) arteries ($\mu = 0.899 \pm 0.473$) did not produce significantly different weights when accounting for Bonferroni correction.

### Computational effort

Preparing the retina segment bags is done dynamically on the CPU, the MIL network utilizes the GPU. On an NVIDIA GTX 2080 Ti, the Kaggle pretraining ran for 10 h 30 min. Training the MIL model took 44 min and allocated a maximum of 2.8 GB of graphics memory.

## Discussion

Our results show that a properly pre-trained multiple instance learning algorithm that processes high resolution CFP images allows for discrimination of healthy controls and patients with PAD. Even though the achieved accuracy leaves some room for improvement, we consider it to be promising given that the majority of eyes (59 out of 101) in the disease class correspond to mild, asymptomatic cases, which represent the first and earliest stage of PAD.

Saliency maps, which can be computed by Grad-CAM^24^ or many other methods, are widely used for visualizing image regions that are particularly relevant for the final classification. These methods can be used with a wide range of neural network architectures, including ones without an attention mechanism, and often achieve a high spatial resolution. In our work, we instead visualized the attention weights, which are an integral part of our multiple instance learning approach. Unlike saliency maps, which are constructed as post-hoc explanations with certain limitations on their interpretability^25^, these attention maps directly affect the network’s decision making, and we found their spatial resolution to be sufficient for distinguishing between the major anatomical structures in our images.

Both our visualization of the attention maps and the statistical analysis of the different anatomical patches confirm our hypothesis, that retinal vascular structures are highly important for the detection of PAD on CFP images. Comparison of the different anatomical structures in Table 3 revealed that the temporal arcade and the optic disc are of particular relevance for CNN based PAD diagnosis based on CFP images. The fact that attention was more significantly increased in patches showing the temporal arcade than in ones showing the nasal arcade does not necessarily imply a smaller involvement of the nasal arcades, but could also reflect the fact that our images only showed a relatively small part of them (Fig. 2).

**Figure 6:**
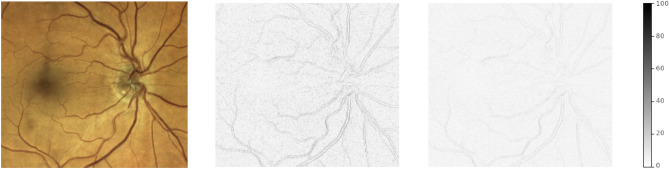
Example of difference maps between the original examination (left) and two down-sampled versions with the resolutions 299 px (middle) and 799 px (right), respectively. Absolute intensity differences (original intensity range: [0, 255]) are color coded in grayscale. In the middle image, differences are mainly seen in the areas of the vessel walls, reflecting a loss of detail in these regions at the lower resolution. Differences are strongly reduced in the picture downsampled to 799 px, hence, anatomical details are better preserved.

We hypothesize that the detected underlying structural imaging biomarkers are associated with general atherosclerosis and, hence, this deep learning approach could aid detection of other atherosclerotic cardiovascular diseases. This hypothesis is further supported by recent results from Chang et al., who were able to predict atherosclerosis using a single Xception model that had been trained on a dataset of 15,408 retina images^7^. The main technical difference between our approach and theirs is that our architecture exploits a higher resolution of the retinal photograph and can account for fine details which are lost when downsampling to a resolution of $299\times 299$ pixels, which is recommended for the Xception network. Our results indicate that using the full resolution achieves a higher accuracy than a reduction to 299 px, and that a resolution between 799 px and 999 px achieves the best results on our dataset. This observation can be explained by two factors: The difference maps in Fig. 6 illustrate that down-sampling removes information from the vessel edges to a much greater extent at small resolutions (299 px) than at higher ones (799 px). This explains the reduced performance for the lowest resolutions as we suspect that atherosclerosis is mainly visible around the vessel boundaries. On the other hand, recent works of Geirhos et al.^26^ and Sheikh et al.^27^ show that CNNs tend to be biased towards image textures and overfit to image details. Our down-sampling to $799 \times 799$ px acts as a smoothing filter and counteracts this source of overfitting. Insights from our visual and statistical analysis of attention weights are in agreement with the gradient maps calculated from the Xception network by Chang et al., which also indicate that the disease markers are most prominent around the optical disc and the retinal arcades.

Our current dataset has some limitations concerning its size and representation of the higher Fontaine stages. In the future, we hope to acquire a larger and more balanced dataset, on which we expect our machine learning approach to yield a further increased precision and recall. When a larger dataset becomes available, the AlexNet component that is used for feature extraction in our current implementation could be replaced by a more complex CNN.

## Conclusions

Our work presents encouraging initial results on detecting peripheral artery disease (PAD) on color fundus photographs with a deep neural network architecture that has been chosen to process images at a high spatial resolution, in order to preserve subtle changes in vascular structures. Our results provide valuable insight about an ocular involvement in PAD and support the idea that systemic cardiovascular disease can be diagnosed from changes in the retinal vasculature. Ablation studies highlight the importance of pre-training on ophthalmic medical images, and working with the primary dataset at a sufficiently high image resolution. Visualization and statistical analysis of attention weights suggest that successful distinction between patients with PAD and healthy controls is enabled by alterations around the optic disc and the temporal retinal vascular arcades.
